# Cultural factors that affected the spatial and temporal epidemiology of kuru

**DOI:** 10.1098/rsos.160789

**Published:** 2017-01-11

**Authors:** J. T. Whitfield, W. H. Pako, J. Collinge, M. P. Alpers

**Affiliations:** 1 MRC Prion Unit and Department of Neurodegenerative Diseases, UCL Institute of Neurology, National Hospital for Neurology and Neurosurgery, Queen Square, London WC1N 3BG, UK; 2 International Health Research, Curtin University, Room 108, Shenton Park Campus, GPO Box U1987, Perth, Western Australia 6845, Australia; 3 Papua New Guinea Institute of Medical Research, PO Box 60, Goroka, Eastern Highlands Province, Papua New Guinea

**Keywords:** epidemiology, cannibalism, transumption, kuru, prion, mortuary rites

## Abstract

Kuru is a prion disease which became epidemic among the Fore and surrounding linguistic groups in Papua New Guinea, peaking in the late 1950s. It was transmitted during the transumption (endocannibalism) of dead family members at mortuary feasts. In this study, we aimed to explain the historical spread and the changing epidemiological patterns of kuru by analysing factors that affected its transmission. We also examined what cultural group principally determined a family's behaviour during mortuary rituals. Our investigations showed that differences in mortuary practices were responsible for the initial pattern of the spread of kuru and the ultimate shape of the epidemic, and for subsequent spatio-temporal differences in the epidemiology of kuru. Before transumption stopped altogether, the South Fore continued to eat the bodies of those who had died of kuru, whereas other linguistic groups, sooner or later, stopped doing so. The linguistic group was the primary cultural group that determined behaviour but at linguistic boundaries the neighbouring group's cultural practices were often adopted. The epidemiological changes were not explained by genetic differences, but genetic studies led to an understanding of genetic susceptibility to kuru and the selection pressure imposed by kuru, and provided new insights into human history and evolution.

## 1. Introduction

Surveillance of kuru ceased in September 2012 after 55 years of fieldwork first instigated by Carleton Gajdusek and Vincent Zigas in 1957 [1] and completed by a collaboration between the UK MRC Prion Unit and the Papua New Guinea Institute of Medical Research. This paper examines the known epidemiology of kuru and provides answers to two of the remaining questions that had been identified about spatial and temporal epidemiological differences in kuru [2,3].

By examining previous studies about the cultural reasons for the traditional mortuary rites [4–8], the origin and spread of kuru by the anthropologists Robert Glasse and Shirley Lindenbaum [9,10], data on the epidemiology of the disease [2,11–15], recently published genetic data [16–18] and new ethnographic data on the traditional mortuary rites of the kuru-affected region [3,19,20], we were able to answer the two principal unresolved epidemiological questions.

These questions are, firstly, why the epidemic spread the way it did from the village of Uwami in the Keiagana linguistic group near the edge of the kuru-affected region and, secondly, why there have been no cases of kuru north of a line across the centre of the kuru-affected region since 1985, when the last case to the south of the line was in 2009. Thirdly, we answered the question as to what cultural and social group principally determined a family's practices during traditional mortuary feasts.

## 2. History of endo- and exocannibalism in Papua New Guinea and the kuru-affected region

We use the term transumption rather than cannibalism to describe the traditional mortuary practices of the kuru-affected region, to which contemporary members of these societies give such good witness, as:

the mortuary practice of consumption of the dead and incorporation of the body of the dead person into the bodies of living relatives, thus helping to free the spirit of the dead. [21, p. 14]

Transumption was a religious practice with associated rituals and was performed out of love and respect for the deceased and their family. It is in distinct contrast to Western perceptions of cannibalism, which can be seen as an offensive term when used in this context. The bodies of the dead were placed in the finest sepulchres that the people had available—their own bodies [20]. Unfortunately, the practice of transumption was the means of transmission of the fatal neurodegenerative disease kuru among these highland people of Papua New Guinea.

It is important to note that the practices of endocannibalism and exocannibalism have been reported worldwide and are not exclusive to Papua New Guinea [22–24]. Although in this paper we focus on transumption, we note here that the consumption of the dead, in the form of mumia (material from dried corpses used in the preparation of medicines between the twelfth and nineteenth centuries in Europe) and fresh blood [25], was an enduring aspect of Western medicines until the recent advent of modern medicine. The practices of blood transfusion and organ transplantation could technically be described as cannibalism, but the use of the term for these modern life-saving Western medical practices would be equally offensive and inappropriate.

Lindenbaum [26] has extended the accepted categories of endocannibalism, exocannibalism and sacrificial, medical and survival cannibalism to include psychopathology, autophagy, placentophagy and innocent anthropophagic practices. Evidence of worldwide anthropophagic practices has been well documented by anthropologists and archaeologists [22–24,26–30]. Genetic evidence supports the hypothesis that past epidemics of prion diseases occurred in humans in prehistoric times transmitted by anthropophagy [16,17].

Anthropophagic practices in Papua New Guinea were recorded by early government officials and anthropologists [31–37].

**Figure 1.**
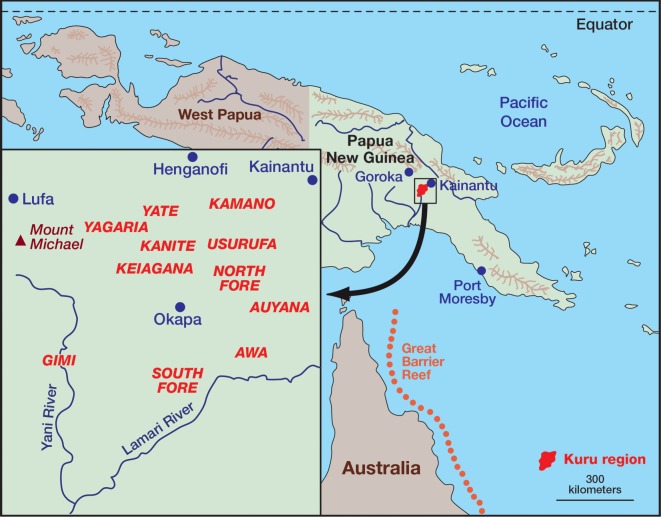
Map of Papua New Guinea with an insert showing the linguistic groups of the kuru-affected region.

There were reports from the early government patrol officers about transumption in the kuru-affected region (figure 1) [38–40]. The first anthropologist to write about transumption in the kuru region was Berndt [4], who wrote that the practice of transumption was connected to the fertility of the ground, and proposed the idea that transumption may have been practised because of protein deficiency. Robert Glasse and Shirley Lindenbaum investigated the distribution of the body during transumption and identified differences between linguistic groups in the distribution of the brain [5]. Robert Glasse [5] wrote that the dead were eaten for gastronomic reasons. Lindenbaum [41] highlighted the importance of the role of transumption in fertilization and regeneration, and pointed out that the body was primarily divided up between the matrilineal kin of the deceased. Sorenson and Gajdusek [6] wrote that traces of the consumed remained in the living. Early investigators were faced with resistance from the indigenous people to the divulgence of their religious beliefs to outsiders and hence they were unable to elicit detailed information about these occult practices. From the published literature, it was clear that there were differences in the practices and ideas about transumption in the kuru-affected region. Further investigation was required to establish the specific details of these differences and to determine whether they were responsible for the changing spatio-temporal epidemiological patterns of kuru.

## 3. The epidemiology of kuru

### 3.1. A history of data collection

Scientific investigations were initiated by Carleton Gajdusek and Vincent Zigas in 1957 [1,42], and continued with the assistance of many fieldworkers and by the establishment of the kuru epidemiological database at the National Institutes of Health in Bethesda, USA by Michael Alpers and colleagues. From 1977 Michael Alpers, then the newly appointed Director of the Papua New Guinea Institute of Medical Research, supervised the epidemiological field surveillance of kuru until its demise in 2012 [2,43]. The surveillance was reinforced in 1996 by collaboration with the Prion Disease Group at St Mary's Hospital in London, later to become the UK Medical Research Council (MRC) Prion Unit, led by Professor John Collinge [44]. This initiative was a response to the epidemic of bovine spongiform encephalopathy (BSE) in the UK and concerns that it might cause disease in humans by consumption of infected cattle [44]. Indeed this concern was realized with the outbreak of variant Creutzfeldt–Jakob disease (vCJD) in the United Kingdom [45] caused by the transmission of BSE to humans [46,47]. While this was thought to pose a significant threat to public health in the UK and elsewhere [48], thankfully the outbreak has been limited to date to around 230 cases; however, recent studies have suggested that many more in the UK population may be silently infected and it is unknown how many of these will develop the disease in the future [49]. Kuru continued into this century and the last three cases were recorded in 2003, 2005 and 2009. It was decided to cease kuru surveillance in September 2012 as the epidemic had essentially ended.

### 3.2. Key epidemiological points about kuru

**Figure 2.**
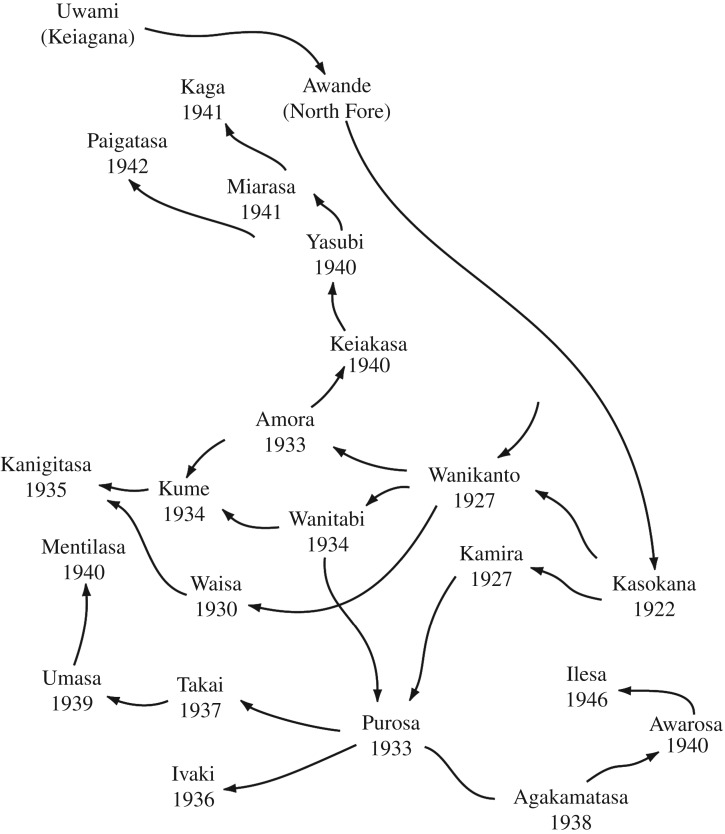
The figure shows the spread of kuru from Uwami in the Keiagana to Awande and Kasokana in the North Fore and its subsequent spread throughout the South Fore. Figure derived with permission from Mathews, 1971 [50]. Slight modifications have been made: Uwami has been added as the source of kuru and the spelling of Kanigitasa changed to its current form.

**Figure 3.**
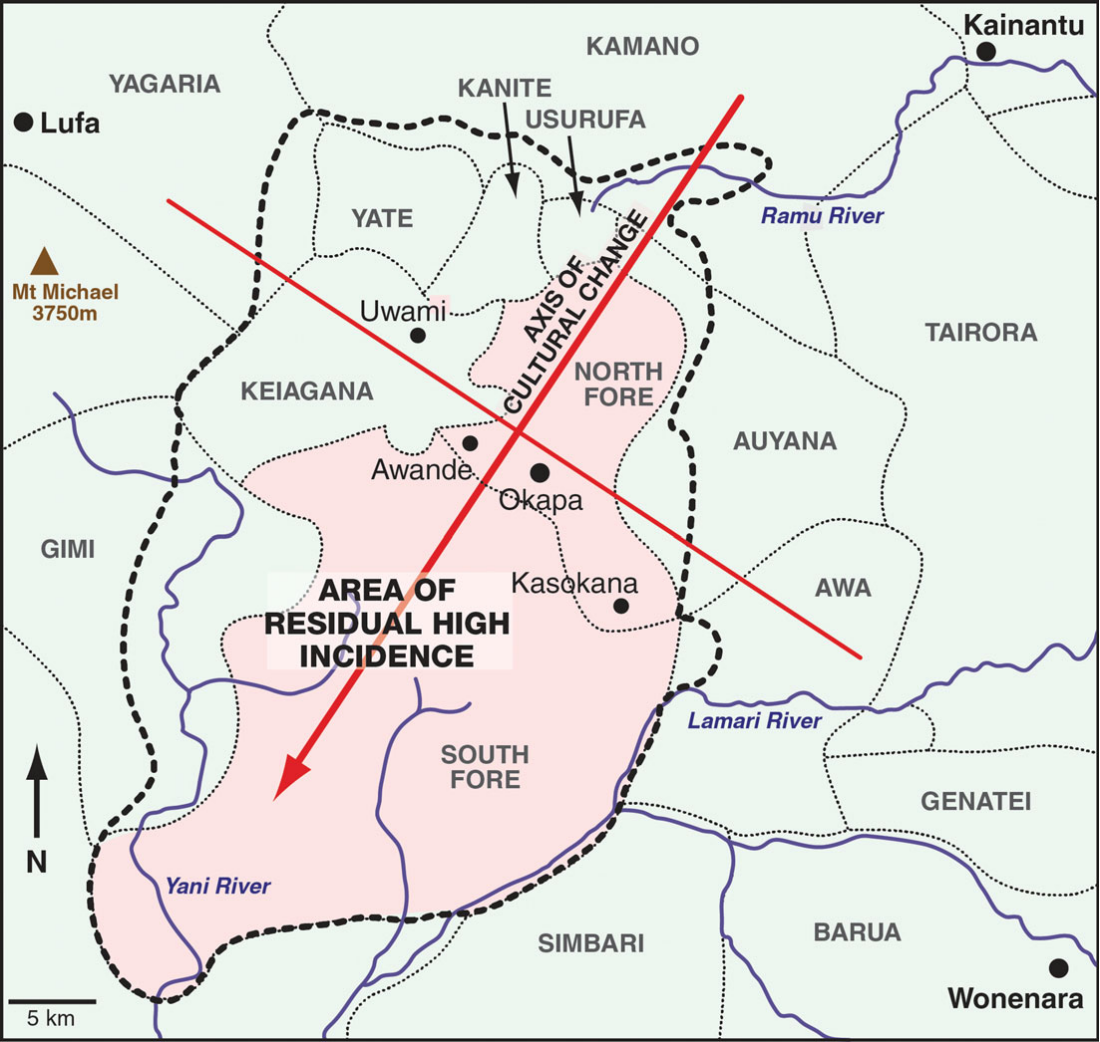
The line drawn across the centre of the kuru-affected region perpendicular to the axis of cultural change separates the area of residual high incidence to the south and low incidence to the north. The kuru-affected area is shown within the black dotted line.

Kuru was found only in a remote area in the southern part of the Eastern Highlands of Papua New Guinea (figure 1). It was confined to the Fore linguistic group and nine contiguous linguistic groups with whom they intermarried. According to oral history, kuru started in the village of Uwami, in the Keiagana linguistic group, and spread from there into the North and then South Fore, with minimal spread within the Keiagana and other linguistic groups (figure 2). The spread of kuru from the Keiagana 100 years ago is well documented [9,10,41,50]. Over 80% of cases occurred in the Fore: over 60% in the South Fore and a further 20% in the North Fore. The peak incidence of kuru was in the South Fore, near the Lamari River, on the far side of which were the Simbari and Barua people of the Anga language groups, where there was no kuru; in the north and west of the kuru region the incidence gradually declined with increasing distance from the Fore (figure 3) [51].

**Figure 4.**
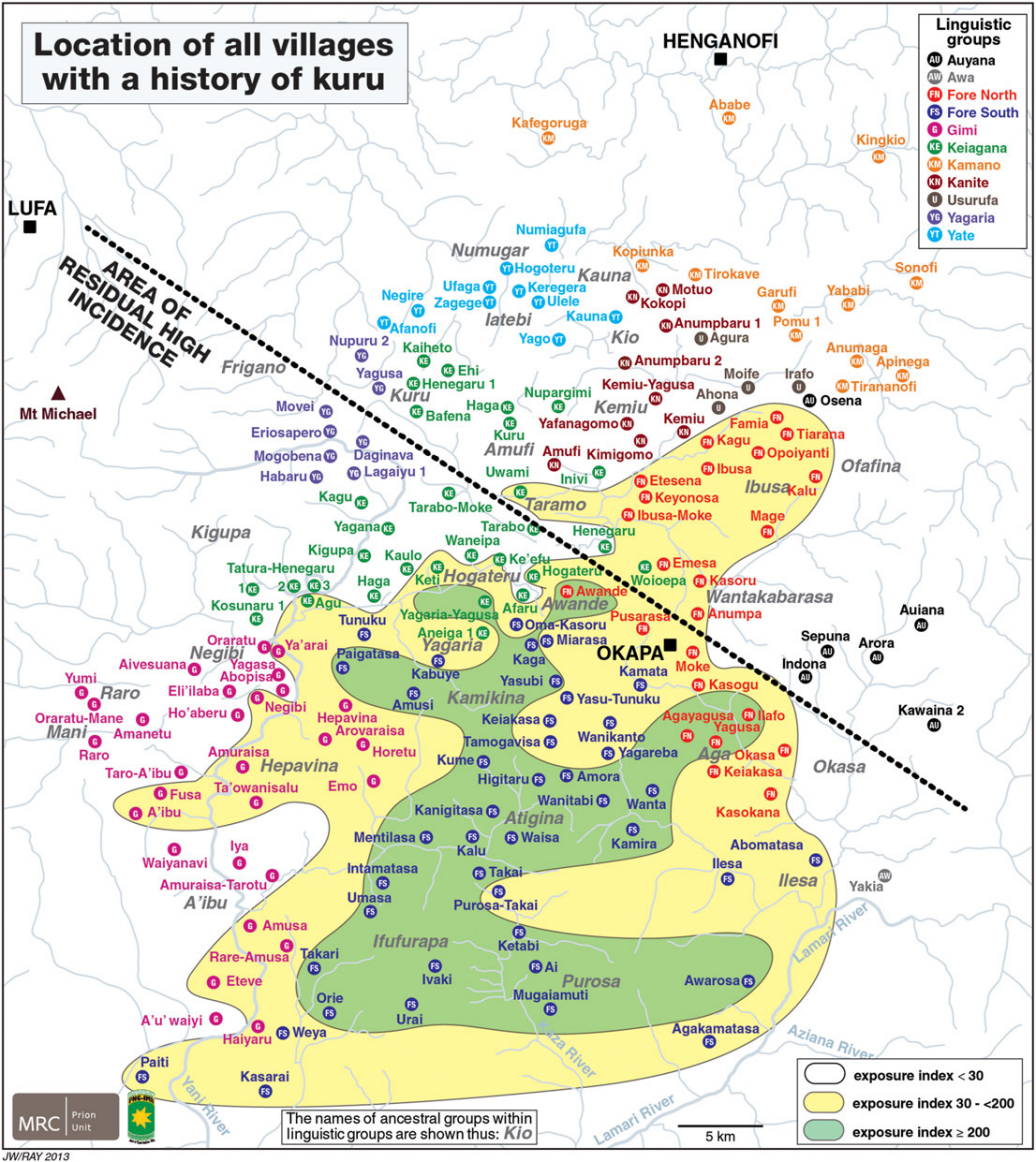
The map shows all villages with a history of kuru. The black dotted line denotes the region of residual high incidence to the south of the line. The coloured areas demarcate the range of EI in villages of the kuru-affected region. The area of highest incidence has an EI of greater than or equal to 200, the area surrounding this an EI of 30 to less than 200, and the peripheral areas an index of less than 30. Based on a figure from Alpers and Kuru Surveillance Team, 2005 [2].

In figure 4, we show the exposure index (EI) for the kuru-affected region on a map with the location of all the villages that had a history of kuru. The area of highest incidence has an EI of greater than or equal to 200, the area surrounding this an EI of 30 to less than 200 and the peripheral areas an index of less than 30. The EI for each village of the North and South Fore and the kuru-affected regions of the Gimi and Keiagana was defined as the total number of deaths from kuru since the beginning of 1957 divided by the estimated village population in 1958, times 1000. For villages in the peripheral areas with fewer kuru deaths the EI was calculated for each of the linguistic groups based on the number of deaths and the estimated kuru-affected population in each linguistic group. It is noteworthy that communities with the highest exposure/incidence cluster around the central South Fore and can be encircled by a single line without any outliers [18]. The communities with intermediate exposure/incidence surround the high exposure cluster and can be encircled by a single line without any outliers. All communities with the least exposure/incidence occupy the remainder of the kuru-affected region peripheral to the high and intermediate zones.

Medical and epidemiological investigations of a still-expanding kuru epidemic started in 1957 within a few years of the first contact between the Fore people and the Australian Administration. The epidemiological features and changing epidemiological patterns have been described from the beginning of kuru investigations and regularly reviewed [2,8,11,12,14,15,42,43,52,53]. Adult females (over 70% of cases) and adolescents and children (about equal numbers of males and females) were those principally affected by kuru, with, on first investigation, no cases in adult males. The proportion of kuru patients who were adult males was 2% by the fourth year of investigation and gradually increased with time. The age of kuru patients at onset ranged from 4 years to over 70 years. Kuru was familial, but was as common among wives as among sisters and daughters. The first kuru victims in a community were often women who had come as wives from a previously affected village, and kuru could spread to a previously unaffected village if an emigrant wife from there developed kuru elsewhere [51].

**Figure 5.**
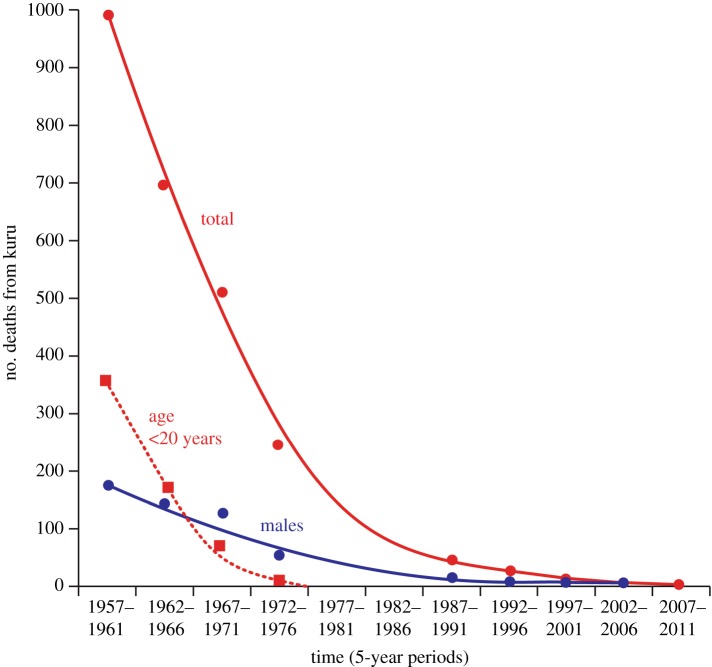
Graph showing the total number of deaths from kuru from 1957, when investigations started, till 2011. The last recorded case of kuru was in 2009. The numbers of deaths in those aged less than 20 years and among males are also shown. By the end of the epidemic there were equal numbers of male and female cases from childhood exposure, all aged in their late 50s or early 60s; since transmission ceased in the 1950s, these cases had incubation periods of 50 years or more.

The epidemic began to decline in the 1960s, and the epidemiological patterns of kuru showed changes from the early 1960s, with progressive disappearance of the disease in the younger age groups and a contraction in the area of high incidence. It has since been established that none of more than 2700 recorded cases of kuru were born after 1959: this birth cohort has grown up completely free of kuru. Cases continued to occur, with decreasing incidence and increasing mean age at onset, after 1960. The mortality from kuru has declined from over 1000 in the first five years of investigation (1957–1961) to 2 in the period 2002–2006 [51] and 1 in the last 5 years studied (2007–2011; figure 5).

By 1974, there were no deaths from kuru aged under 20 years; all those dying from kuru since 1994 have been aged over 45, and since 1999 over 50 years. Since 1986, all cases of kuru have been in the southern area of residual high incidence, mostly in the South Fore, with adjacent communities in the Gimi, Keiagana and North Fore linguistic groups (M.P.A. 2016, unpublished data from the kuru database). The last case of kuru in the Keiagana was in 1987, in the Gimi in 1993 and in the North Fore in 1995. There have been 14 cases since 1996, eight males and six females, aged 48–64 years.

The origin of kuru was postulated to be a sporadic case of CJD [54], a disease which occurs at random in all human populations and is thought to represent the rare spontaneous production of prions [55]. More recently, prion strain typing has confirmed that kuru prion strains are indistinguishable from those causing sporadic CJD, consistent with this origin of kuru [56].

Once kuru was proved to be experimentally transmissible by inoculation of primates [57], the changing epidemiological patterns were explained by the known practices and recent cessation of transumption, thus demonstrating that transumption was the mode of transmission of the infective agent [13,58]. The restriction of kuru to a remote area of the Eastern Highlands depended on the chance combination of a sporadic case of infectious human prion disease in a community practising transumption as the preferred means of disposal of the dead. Women and children were the main participants in transumption, and this explained the high incidence of the disease among these groups. The initial spread of kuru to communities through affected wives depended on the key role played by affines in the practice of transumption. Where there was no intermarriage with the Fore, as in the Anga and Pawaian groups, there was no kuru. The decline in kuru incidence was explained by the cessation of the mortuary practice of transumption, summarily banned in the 1950s by the Australian administrative authorities. Though the cohort born since 1960 were growing up free of kuru, their mothers continued to come down with kuru and die of it; vertical transmission was thus excluded [13]. Though most of the epidemiological features of kuru were readily explained, there were remaining questions. To try and answer these, more recent genetic and mortuary practice studies were undertaken.

### 3.3. Remaining questions

The first question of interest was why kuru spread the way that it did from the village of Uwami in the Keiagana, which is near the edge of the kuru region (figures 2 and 3). It spread from there to the south and east into the Fore region. Kuru became common in the North Fore, and reached a high level of incidence in the South Fore, and yet faded out in the Keiagana and other linguistic groups to the north and west [11]. The spread of kuru has been documented from its source in Uwami to Awande, and Kasokana, and then north into the rest of the North Fore and throughout the South Fore [9]. It was also noted that the North Fore were less enthusiastic about eating those who died of kuru than the South Fore [58]. Those communities to the northwest (figure 4), where kuru faded out, intermarried with the Fore, and Fore women and their children who migrated into this region developed kuru. Women who migrated into the Fore often remained free of the disease, but their children developed kuru [13]. The possible reasons for the historical spread of kuru were raised by early investigators, who considered that genetics, chance or mortuary practices could provide the explanation. This paper answers this long-standing question.

There has been an axis of social and cultural change within the kuru-affected region since the late 1940s, and a line in the centre of the region perpendicular to this axis demarcates the area of residual high incidence of kuru (figure 3) [2]. As noted above, all deaths from kuru from 1986 onwards have been in the area south of this line, and have occurred mainly in the South Fore, with a few cases in the North Fore (in villages south of the line) and in the Gimi and a single case in the Keiagana. This difference had no obvious epidemiological explanation. Why in those villages to the north of the perpendicular cultural line had there been no deaths from kuru since 1985, 24 years before the last recorded death in the South Fore? Although the central part of the North Fore came under government control at least 5 years before the South Fore and thus ceased transumption several years earlier, the gap of more than 20 years remained unexplained.

The Gimi linguistic group was the last part of the kuru-affected area to come under Australian administrative control and thus the last area to cease practising transumption. The last death from kuru in the Gimi occurred in 1993 and the last two in the South Fore in 2005 and 2009; this raised the questions as to why kuru disappeared in the Gimi before the South Fore and why there was a 16-year difference between the two areas [2]—it was a 12-year difference in 2005 when the observation was first made.

The areas of zero and residual high incidence also presented another problem to investigators. There were North Fore villages in the areas of zero and residual high incidence (figures 3 and 4). This raised the question as to what was the principal factor that determined cultural practices, in particular the mortuary practices, in a village. Was it membership of the linguistic group, dialect group, ancestral group, the village, the clan, the hamlet or the household that determined cultural behaviour at mortuary feasts? The village was an important social unit, but consisted of a changeable association of individual hamlets. The attitude of the head of each household had a large influence on the behaviour of its members. The ancestral group was a traditionally named group of historical and social significance that was not much invoked, however, in daily life. The dialect often, but not always, had a traditional name but there was no organization or structure that defined the people who spoke it. Language was an important component of these cultures, but in this part of the highlands there was no political structure or traditional name that united people who spoke a common language, nor would this have had much meaning where language boundaries merged through a dialect chain. Though all these groups had deep cultural significance—either implicitly or explicitly—their influence on the details of any particular cultural practice or behaviour varied considerably.

## 4. Methodology

### 4.1. Interaction with and support of participating indigenous communities

The anthropologist in the team (J.T.W.) and field staff established and maintained the participation of communities through discussions with community leaders, communities, families and individuals. In this case, where there were no therapeutic benefits available to those suffering from the disease under study, it was important to achieve some form of human, social or structural development in the communities involved. Indeed, there was already a legacy of obligations between investigators and the people of the kuru-affected region [59].

The needs of participating communities were identified through discussions, and the project facilitated the communities' chosen modes of development. The project sourced UK charitable or overseas aid funding for community development projects and oversaw the establishment of water supplies, schools, malaria control along the Lamari Valley and a village birth attendant programme.

The study involved 14 indigenous field staff from the kuru-affected region, logistical and administrative support of the Papua New Guinea Institute of Medical Research and scientific analysis by the MRC Prion Unit in the United Kingdom.

### 4.2. Ethnographic methodology

The data presented include field notes and transcriptions which were collected between 1996 and 2010. The following numbers of informants who had participated in or witnessed transumption were interviewed: from the South Fore, 68 men and 20 women (from 20 different villages and 22 different clans); and from the North Fore, 16 men and nine women (from five villages and 11 clans). A further 72 men and women were interviewed from the Gimi, Keiagana, Kanite, Yagaria, Yate, Kamano, Auyana, Awa and Usurufa linguistic groups. The gender imbalance is very apparent in the South Fore, as many villages have no elderly women survivors of the kuru epidemic. The elderly people interviewed had all participated in or witnessed transumption during childhood. Male children participated with their mothers until the age of between 6 and 8 years, when they underwent their first initiation and lived with the older males in the warriors' house. This meant that the males and females interviewed had nearly equal childhood witness to transumption.

We used a phenomenological methodology for the collection of data. Here, ‘phenomenological’ means the most direct expression achievable, through translation and interpretation, of the beliefs, concepts and phenomena described by the people in their own terms.

Overall, the mortuary rites of the linguistic groups of the kuru-affected region shared the same practice of transumption and other means of disposing of the dead. Detailed descriptions of the mortuary rites have been written up elsewhere [3,19,20].

## 5. Results

### 5.1. Explanation for the origin and spread of kuru

Robert Glasse and Shirley Lindenbaum used local oral history to trace the origin of kuru to Uwami in the Keiagana linguistic group around the turn of the twentieth century [7,9]. They also recorded its subsequent spread to Awande, Kasokana and throughout the South Fore (figure 2). Kuru was soon established in most North Fore villages and somewhat later in the southern South Fore, and was reported as reaching Paiti in the southwest corner of the South Fore only at the start of the 1950s.

Considering the uniform worldwide incidence of sporadic CJD, one to two cases per million population per annum [60], it was inevitable that a case would at some time occur in the area where transumption was practised and, if consumed, could then cause an outbreak of a transmissible prion disease. Once this rare event had occurred and the body was eaten, others would eventually become infected and develop kuru. When they were consumed in turn by their affines from other communities, the spread of kuru would have continued and the epidemic would have gradually expanded and amplified. The participation in transumption by the affines and other guests allowed kuru to spread throughout the Fore region.

Early in the kuru epidemic those who died of kuru were consumed in the Keiagana, Kanite, Usurufa, Yagaria, Yate and Kamano, but this quickly changed when the people there concluded that kuru was contagious, and they ceased to eat the corpses of those who died of kuru. In the Awa those who died of kuru were never disposed of by transumption. Transumption was not an accepted cultural practice among the Auyana adjacent to the kuru-affected region, and in these cases transmission occurred when affines attended feasts in a Fore community where they had marriage ties. By contrast, the Fore and adjacent Gimi incorporated kuru into their sorcery-based nosology and continued rigorously to dispose of their kuru dead by transumption. Thus, the mortuary practices provide a sufficient explanation for the way kuru spread from its initial focus, and subsequent differences in these practices explain why the epidemic faded out in the Keiagana and other linguistic groups to the north and west but amplified and expanded in the Fore.

Other cultural factors associated with transumption also contributed to these differences. In practice, all the women in the North and South Fore took part in transumption, except for the occasional one who was prohibited by her husband. The women and children ate brain, spinal cord and internal organs in almost every community. In contrast, in the Keiagana, Kanite, Kamano, Yagaria, Yate, Usurufa, Awa and Gimi only older women (with two to four children) participated in the transumption of the brain, spinal cord and internal organs. Children in these groups were not allowed to consume brain, spinal cord or internal organs. Thus nearly all the women and children in the Fore had the chance of being exposed to the infective agent, in contrast to the other linguistic groups, where, in the main, only older women were exposed. Children from these linguistic groups could still have become infected since they had marriage ties with the Fore, with the possibility of exposure during Fore mortuary feasts. Nevertheless, only adult cases of kuru were recorded from the Kamano, Usurufa and Auyana linguistic groups and though all other groups had adolescent cases (aged 12–19 years), there were no cases in children (aged less than 12 years) in the Kanite, Yagaria, Yate and Awa linguistic groups or in the Keiagana villages north of the central cultural dividing line (figures 3 and 4; M.P.A. 2016, unpublished data from the kuru database).

Even before investigations began into kuru, the axis of cultural change from the northeast across the kuru-affected region (figure 3) brought changes that affected the spatial and temporal distribution of kuru. The first government patrols entered the northernmost affected areas in 1947 and soon after police posts were established and village officials appointed. North Fore men were working in agricultural stations in Kainantu and Aiyura as early as 1952 and Papua New Guinean missionaries were already living in the South Fore in the same year. The Lutheran Mission was established at Tarabo in the Keiagana in 1949. A police post was established in Moke in 1951 and the government station of Okapa near Moke village in 1954. The government officials and missionaries admonished the local people for practising transumption and they quickly conformed to the intruders' wishes. Because of this axis of social change, it is likely that kuru was already on the decline in the Kamano, Usurufa, Kanite, Yate, Yagaria, northern Keiagana and northern North Fore before investigations began in 1957.

### 5.2. How mortuary practices affected the spatial and temporal epidemiology of kuru

Differences in mortuary practices also explain the striking epidemiological gap of 24 years between the last death from kuru in the area north of the line demarcating the area of residual high incidence and that south of the line.

The South Fore population and the populations of the southern North Fore villages (including Awande and Kasokana) shared the same mortuary practices. They had the most exposure to the infective agent since they consumed those who died of kuru. They also had the largest number of people exposed, as women of all age groups and children participated in the transumption of infective tissue. In the main northern part of the North Fore, the women and children were also exposed but, as with the other linguistic groups to the north, they later began to classify kuru as a contagious disease that made transumption a dangerous practice. In the South Fore, the cessation of transumption took place gradually over a decade, with no transmission of kuru taking place after the early 1960s. The effects of the axis of social change meant that, by 1950, the northernmost areas were already starting to comply rigorously with the Administration's wishes to cease transumption and bury their dead, several years ahead of the South Fore. However, the most powerful factor reducing the transmission of kuru had been the earlier decision taken by the people of these cultural groups not to eat their kuru dead. This explained the 24-year difference in the demise of kuru north and south of the central line of demarcation.

### 5.3. The cultural and social group that primarily determined the mortuary practices

Earlier, we noted that there were North Fore villages in the area of residual high incidence although most were in the area where kuru had already ended (figures 3 and 4). A similar division also occurred in the Keiagana. This raised the question as to what were the principal factors that determined cultural practices, in particular mortuary practices, in a village. Language was a critical factor and within a linguistic group we found a general consistency of practice, with only the occasional exception. These exceptions were often carried out by refugees from elsewhere. Importantly, cultural practices were more flexible, or more diverse, in border communities, either between languages or between dialects; for example, a village in a border area might speak one language, but their mortuary practices might be those of the neighbouring linguistic group. The North Fore villages in the area of high residual incidence of kuru had the same mortuary practices as those of the South Fore, rather than those of the majority of North Fore communities. This situation also occurred in the southern Keiagana (in the Hogateru and Yagaria ancestral groups) and in the Hepavina region of the Gimi (figure 4). Thus, the linguistic group was shown to be the primary group that determined behaviour, but at linguistic boundaries a village community, which is the other most influential social group, or even a whole ancestral group such as Hepavina, might choose to follow the cultural practices of their neighbouring linguistic group.

### 5.4. The Gimi question

In the Gimi, those who died of kuru were in general not eaten; however, among the border communities of the Hepavina region of the Gimi, where both the Gimi and Fore languages were spoken, communities consumed those who died of kuru. The mortality from kuru was much higher in the Hepavina region than elsewhere in the Gimi [61] and this was also true for the EI (figure 4).

Kuru finally ended in the Gimi (in the Hepavina) in 1993 and in the North Fore (in villages south of the central cultural dividing line) in 1995. The last cases of kuru were in the central South Fore and showed the equal sex ratio of childhood transmission, with incubation periods stretching beyond 50 years. The unexpected earlier demise of kuru in the Gimi, 16 years before the South Fore, was noted as an epidemiological question that required explanation [2]. However, investigation of this question was not able to provide a satisfactory link between the accounts of specific local mortuary practices and the epidemiological findings. Furthermore, the question was misplaced because the mortuary practices were not in this case determined at the linguistic group level, as our subsequent anthropological investigations have established. There was no Gimi–South Fore dichotomy but, as described in the previous section, the Hepavina Gimi villages that bordered the South Fore adopted the practices of the South Fore. This was also true of the southern North Fore and southern Keiagana. These contiguous villages created with the South Fore an expanded cultural domain with respect to their mortuary practices and the consumption of the kuru dead. Within this domain there is, in fact, a wide range in the years when the last case of kuru in a village occurred, going back well before 1993, and this needs to be investigated across the whole domain. Studies of this extended cultural domain will be undertaken once the final systematic review of all the kuru case records, now in progress, has been completed.

### 5.5. Genetics of kuru

The purpose of the investigation into the genetics of kuru was to understand, in a nearly completed epidemic, genetic susceptibility and resistance, and the genetic effects on the incubation period that were relevant to the vCJD epidemic.

It has been established that heterozygosity for a common prion protein polymorphism (either methionine (M) or valine (V) is encoded at codon 129 of *PRNP* (the prion protein gene)) provides relative resistance to prion infection. MV individuals are less susceptible and, if infected, generally have a longer incubation period. This polymorphism also influences propagation of different prion strain types as the M and V forms of human prion protein preferentially propagate different prion strains according to the conformational selection model [48,62,63].

Among those suffering from kuru, the children were predominantly 129MM homozygous and to a lesser degree 129VV. Older patients had a greater proportion of 129MV genotypes and a lack of 129MM genotypes. This was particularly the case with the elderly female survivors of the epidemic and recent cases of kuru with long incubation periods [64]. While genetic susceptibility was a strong factor in determining individual incubation times and those who were more likely to survive the kuru epidemic, known genetic factors cannot explain the temporal or geographical spread of kuru. The heterozygous genotype at codon 129 is ancient, very common and widespread throughout the Eastern Highlands at broadly similar genotype frequencies [16,18], and therefore cannot have determined the regions most affected.

Contrary to the possibility that pre-existing genetic diversity may have determined the regions affected by kuru, there is some evidence of a population genetic response to the kuru epidemic. In the Purosa valley area, which experienced one of the highest incidence rates of kuru, a completely protective variant of the prion protein gene (G127V) was found in some families [18,65]. The genetic data are most in keeping with the action of strong positive selection on the 127V allele because of selection pressure conferred by the devastating incidence of kuru on the fertile or younger population. Left unchecked by administrative intervention, selection pressure would have persisted and we might speculate that 127V would have continued to rapidly increase in frequency. Consequently, should 127V have become a common and more widespread allele, the population resistance to disease could have limited the spread of kuru. However, the local distribution and low frequency of the variant rules out any significant contribution to the established spatio-temporal epidemiological changes.

The Prion Unit studies suggested that strong balancing selection has influenced the prion protein gene in all populations worldwide, and a rough estimate of the age of the codon 129 polymorphism is 500 000 years. The most probable explanation for this balancing selection is that heterozygosity at the prion protein gene gives resistance to human prion infections, and that populations were exposed to prion infection during the regular consumption of humans in the remote human past [16].

## Data accessibility

The results of this work are based on data from privately recorded interviews about past secret practices and sacred beliefs which were given to us as privileged information about traditional religious practices by senior members of communities in the Papua New Guinea highlands because of the relevance of this information to the epidemic of the disease kuru that afflicted their communities. We cannot disclose the primary data to others without first seeking the permission of the communities involved, and to make these confidential personal accounts publicly available would be a serious breach of trust and the ethical requirements of the study. Data may be sought from the authors with their permission.
